# Comparative Genomics Uncovers Large Tandem Chromosomal Duplications in *Mycobacterium Bovis* BCG Pasteur

**DOI:** 10.1002/1097-0061(20000630)17:2<111::AID-YEA17>3.0.CO;2-G

**Published:** 2000

**Authors:** Roland Brosch, Stephen V. Gordon, Carmen Buchrieser, Alexander S. Pym, Thierry Garnier, Stewart T. Cole

**Affiliations:** 1 Unité de Génétique Moléculaire Bactérienne Institut Pasteur 28 Rue du Dr Roux Paris Cedex 15 75724 France; 2 Laboratoire de Génomique des Microorganismes Pathogènes Institut Pasteur 25 Rue du Dr Roux Paris Cedex 15 75724 France; 3 Veterinary Laboratories Agency Woodham Lane New Haw Addlestone Surrey KT15 3NB UK

## Abstract

On direct comparison of minimal sets of ordered clones from bacterial artificial chromosome (BAC) libraries representing the complete genomes of *Mycobacterium tuberculosis* H37Rv and the vaccine strain, *Mycobacterium bovis* BCG Pasteur, two major rearrangements were identified in the genome of *M. bovis* BCG Pasteur. These were shown to correspond to two tandem duplications, DU1 and DU2, of 29 668 bp and 36 161 bp, respectively. While DU1 resulted from a single duplication event, DU2 apparently arose from duplication of a 100 kb genomic segment that subsequently incurred an internal deletion of 64 kb. Several lines of evidence suggest that DU2 may continue to expand, since two copies were detected in a subpopulation of BCG Pasteur cells. BCG strains harbouring DU1 and DU2 are diploid for at least 58 genes and contain two copies of *oriC*, the chromosomal origin of replication. These findings indicate that these genomic regions of the BCG genome are still dynamic. Although the role of DU1 and DU2 in the attenuation and/or altered immunogenicity of BCG is yet unknown, knowledge of their existence will facilitate quality control of BCG vaccine lots and may help in monitoring the efficacy of the world's most widely used vaccine.


**Yeast**
*Yeast* 2000; **17**: 111–123.

**Research Article**

### Comparative genomics uncovers large tandem chromosomal duplications in *Mycobacterium bovis* BCG Pasteur

Roland Brosch[1], Stephen V. Gordon[1,†], Carmen Buchrieser[2], Alexander S. Pym[1], Thierry Garnier[1] and Stewart T. Cole[1]*

[1] *Unité de Génétique Moléculaire Bactérienne, Institut Pasteur, 28 Rue du Dr Roux, 75724 Paris Cedex 15, France*
[2] *Laboratoire de Génomique des Microorganismes Pathogènes, Institut Pasteur, 25 Rue du Dr Roux, 75724 Paris Cedex 15, France*

**Correspondence to:*

*S. T. Cole, Unité de Génétique Moléculaire Bactérienne, Institut Pasteur, 28 Rue du Dr Roux, 75724 Paris Cedex 15, France.*

*E-mail: stcole@pasteur.fr*

*† Present address:*
*Veterinary Laboratories Agency, Woodham Lane, New Haw, Addlestone, Surrey KT15 3NB, UK.*

Received: 18 February 2000
Accepted: 17 April 2000

### Abstract

**On direct comparison of minimal sets of ordered clones from bacterial artificial chromosome (BAC) libraries representing the complete genomes of *Mycobacterium tuberculosis* H37Rv and the vaccine strain, *Mycobacterium bovis* BCG Pasteur, two major rearrangements were identified in the genome of *M. bovis* BCG Pasteur. These were shown to correspond to two tandem duplications, DU1 and DU2, of 29 668 bp and 36 161 bp, respectively. While DU1 resulted from a single duplication event, DU2 apparently arose from duplication of a 100 kb genomic segment that subsequently incurred an internal deletion of 64 kb. Several lines of evidence suggest that DU2 may continue to expand, since two copies were detected in a subpopulation of BCG Pasteur cells. BCG strains harbouring DU1 and DU2 are diploid for at least 58 genes and contain two copies of *oriC*, the chromosomal origin of replication. These findings indicate that these genomic regions of the BCG genome are still dynamic. Although the role of DU1 and DU2 in the attenuation and/or altered immunogenicity of BCG is yet unknown, knowledge of their existence will facilitate quality control of BCG vaccine lots and may help in monitoring the efficacy of the world's most widely used vaccine.** **Copyright © 2000 John Wiley & Sons, Ltd.**

**Keywords:** **BCG; *Mycobacterium tuberculosis*; tandem duplication; vaccine; comparative genomics; *Mycobacterium bovis*; genome plasticity**

### Introduction

BCG, the 'Bacille de Calmette et Guérin', originally derived from virulent *Mycobacterium bovis*, is one of the oldest and most widely used live vaccines [8]. About 3 billion doses of this attenuated *M. bovis* strain have been used to immunize individuals against tuberculosis worldwide. Although the various vaccination trials have revealed great variability in the protective efficacy of BCG against pulmonary disease [11], there is general agreement that the vaccine is effective against the dissiminated forms, miliary tuberculosis and tuberculous meningitis [4]. Several hypotheses have been proposed to explain this variability, ranging from differences in BCG substrains to environmental and host factors [4]. Indeed, the history of BCG since the original attenuation obtained by Calmette and Guérin is very complex, due to parallel propagation of different cultures *in vitro* over a long period, resulting in genomic modification and adaption to *in vitro* growth, which has led to the appearance of several substrains of BCG [1,2,17].

However, the close relationship of BCG to the other members of the *M. tuberculosis* complex, which share >99.9% identity at the DNA level, enables us to use the complete genome sequence of *M. tuberculosis* H37Rv [9], together with comparative genomics, to elucidate the genetic peculiarities of *M. bovis* BCG and its various substrains. The first genomic differences between *M. bovis* and *M. bovis* BCG Connaught were described by Mahairas *et al.* [16], using subtractive hybridization techniques. Their work revealed three genomic

Copyright © 2000 John Wiley & Sons, Ltd.

regions (RD1–RD3), which were absent from BCG but present in *M. bovis* and *M. tuberculosis*. Closer inspection of these deletions showed that RD1 was consistently absent from all BCG substrains and present in all strains of *M. tuberculosis* and *M. bovis* tested [16]. In contrast, RD2 was only deleted from some BCG substrains, while RD3, corresponding to the prophage phiRv1 [9], was also absent from several virulent *M. bovis* isolates [16]. Complementation of BCG with RD1 has not yet been shown to restore virulence in the animal model, which suggests that the BCG attenuation process may have been more complex than was previously thought [16]. Further whole genome comparisons using bacterial artifical chromosome (BAC) arrays [6,12], or DNA microarrays [2], identified several other differences between *M. tuberculosis, M. bovis* and BCG strains. The majority of these deletions observed in BCG strains were also detected in *M. bovis* strains, indicating that they represented differences between *M. bovis* and *M. tuberculosis*, rather than BCG-specific deletions [2,12]. However, these sophisticated hybridization techniques can only detect deletions in BCG relative to *M. tuberculosis* H37Rv (the template DNA) and cannot, therefore, identify insertions and chromosomal rearrangements.

Thus, in an attempt to characterize the genome of the vaccine strain *M. bovis* BCG Pasteur 1173P2 more precisely, we have used a complementary approach to the previously used BAC arrays for detecting possible insertions and genomic rearrangements. This approach is based on pulsed-field gel electrophoresis of *Hin*dIII restriction fragments from selected *M. tuberculosis* H37Rv and BCG BACs, covering comparable genomic regions [12]. Using this strategy on a genome-wide basis we identified two large rearrangements (DU1 and DU2), which were not detected by other methods [2,6,12,19], which may have an important impact on the biology and immunogenicity of the vaccine strain.

### Materials and methods

### Bacterial strains and plasmids

The *M. tuberculosis* complex strains (*M. tuberculosis* H37Rv, *Mycobacterium bovis* type strain ATCC 19210) were taken from our laboratory stocks, while *M. bovis* BCG substrains (Denmark, Glaxo, Prague, Russia, Japan) were kindly supplied by N. Winter and G. Marchal from the BCG laboratory, Institut Pasteur, Paris. *M. bovis* BCG Pasteur 1173P2 was received as a lyophilized vaccine.

### Genomic DNA preparation

Preparation of agarose-embedded genomic DNA from BCG Pasteur was performed as previously described [5,20]. Plugs were stored in 0.2 M EDTA at 4°C and washed twice in 50 ml Tris–EDTA (pH 8)/Triton X-100 (0.1%) at 4°C for 1 h, then twice in 50 ml restriction enzyme buffer/Triton X-100 (0.1%) for 1 h at room temperature before use.

### Comparative genomics

Overlapping clones from the *M. tuberculosis* H37Rv pBeloBAC11 library [6] and the BCG pBeloBAC11 (*Hin*dIII fragments) and pBACe3.6 (*Eco*RI fragments) libraries [12] were selected on the basis of their endsequences. BAC DNA from these clones was prepared from 40 ml cultures, grown in 2 × YT medium containing 12.5 μg/ml chloramphenicol, as previously described [6]. DNA (100–200 ng) was digested with *Hin*dIII (Boehringer), *Dra*I (Gibco-BRL) or *Xba*I (Boehringer) and restriction products separated by pulsed-field gel electrophoresis (PFGE) on a Biorad CHEF II apparatus using a 1% (w/v) agarose gel, and a pulse of 1 s ramped to 1.6 s for 22 h (*Hin*dIII) or 1 s to 18 s for 29 h (*Dra*I, *Xba*I) at 6 V/cm. Low-range PFGE markers (New England Biolabs) were used as size standards. Insert sizes were estimated after staining with ethidium bromide and visualization with UV light. Agarose gels were treated by the standard Southern method and the DNA transferred via capillary blotting to Hybond-C Extra nitrocellulose membranes (Amersham). DNA was fixed to the membrane by baking at 80°C for 2 h. PCR-derived DNA was labelled with [$\alpha$-$^{32}$P]dCTP using the Prime-It II kit (Stratagene), and purified through P10 columns (Biorad) before use. Hybridizations were performed at 37°C in 50% formamide, as previously described [20]. Membranes were washed for 15 min at room temperature in 2 × SSC/0.1% SDS, then 1 × SSC/0.1% SDS, and finally 0.1 × SSC/0.1% SDS. Results were interpreted from the autoradiograms. Sequencing primers that flanked the junction regions were then designed for PCR and sequencing reactions with the corresponding BCG BAC used as tem-

Copyright © 2000 John Wiley & Sons, Ltd. 

plate, using our in-house *M. tuberculosis* H37Rv database.

### PCR Analysis

Primers used in PCR reactions are listed in Table 1. Reactions contained 5 μl of 10 × PCR buffer (100 mM $\beta$-mercaptoethanol, 600 mM Tris–HCl pH 8.8, 20 mM $MgCl_2$, 170 mM $(NH_4)_2SO_4$), 5 μl 20 mM nucleotide mix, 0.2 μM each primer, 10–50 ng template DNA, 10% DMSO, 0.5 units *Taq* polymerase (Gibco-BRL) and sterile distilled water to 50 μl. Thermal cycling was performed on a PTC-100 amplifier (MJ Inc.) with an initial denaturation step of 90 s at 95°C, followed by 35 cycles of 30 s at 95°C, 1 min at 56°C, and 4 min at 72°C.

### Sequencing reactions

Sequencing reactions were performed as previously described [6]. For clones isolated from the pBeloBAC11 library, SP6 and T7 primers were used to sequence the ends of the inserts, while for pBACe3.6 clones (XE-clones), vector-derived primers were used (Table 1). PCR products were purified prior to sequencing using the QIAquick PCR purification Kit (QIAGEN). Reactions were loaded onto 48 cm, 4% polyacrylamide gels and electrophoresis performed on ABI373A or 377 automated DNA sequencers (Applied Biosystems) for 10–12 h. Reactions generally gave between 400–700 bp of readable sequence.

### Bioinformatics

Sequence data were transferred from the ABI automated sequencer to DEC-alpha workstations (Digital) and edited using the TED software from the Staden package. Edited sequences were compared to the in-house *M. tuberculosis* database (H37Rv.dbs) to determine the relative positions of the end-sequences on the complete genome sequence. Using this method, the sizes of *M. tuberculosis* H37Rv and *M. bovis* BCG Pasteur BAC clones were predicted and compared to those estimated by PFGE analysis of the same clones. *In silico* restriction enzyme digestions of selected genomic regions were performed using the Display and Analysis programs (DIANA, Artemis) from the Sanger Centre, Cambridge, UK (available via **http://www.sanger.ac.uk/Software/**).

Table 1. Primers for PCR and sequencing

| | |
|---|---|
| Junction DU1 | |
| TB16.0F: | GAG CCA ACG ATG ATG ATG ACC |
| TB16.5F: | GGT CAC GGT CGG TGT CGT C |
| TB4398.7R: | CAG AAC TGC AGG GGT GGT AC |
| Junction DU2A | |
| TB3689.5F: | CTA GTT GTT CAG CCG CGT CTT |
| TB3591.0R: | ACC GGG GTG TCG GCC AGT T |
| TB3689.9F: | TCG CGG CCA CCG TGC GTA A |
| TB3591.5R: | GGC GCC TAT GAC TGA TAC CC |
| Junction DU2B | |
| TB3608.0F: | GAA CAG GGT CGC GGA GTC T |
| TB3672.0R: | TCG AGG AGG TCG AGT CCT GT |
| TB3671.7R: | GGG TTC ATG AGG TGC TAG GG |
| RvD5-detection primers | |
| RvD5-intF: | GGG TTC ACG TTC ATT ACT GTT C |
| RvD5-intR: | CCT GCG CTT ATC TCT AGC GG |
| BCG-Pasteur specific deletion | |
| Rv1769F: | GTGGAGCACCTTGACCTGAT |
| Rv1769R: | CGTCGAATACGAGTCGAACA |
| Hybridization-probe for DU1 | |
| TB4411.0F: | CCG GCC ACT CAC TGC CTT C |
| TB0.3R: | ACG GTA GTG TCG TCG GCT TC |
| Hybridization-probe for DU2 (probe 3675) | |
| TB3675.0F: | CCA ACA CCG TCA ACT ACT CGA |
| TB3675.5R: | ATC GCA GAA CTC CGG CGA CA |
| Sequencing of the *dnaA–dnaN* region | |
| TB1.2F: | CGA TCT GAT CGC CGA CGC C |
| TB1.5F: | TCC GTC AGC GCT CCA AGC G |
| TB1.8F: | GTC CCC AAA CTG CAC ACC CT |
| TB2.2R: | AAT CCG GAA ATC GTC AGA CCG |

### DNA sequence Accession Nos

The nucleotide sequences that flank each junction region in *M. bovis* BCG Pasteur have been deposited at the EMBL database. Accession Nos for junctions JDU1, JDU2A and JDU2B are AJ249608, AJ249609 and AJ249610, respectively. Accession Nos for the *dnaA–dnaN* spacer region on clones X592 and X703 are AJ249809 and AJ249810, respectively. The Accession No. for *M. tuberculosis* H37Rv-deleted RvD5 region present in *M. bovis* BCG Pasteur (BAC X094) is AJ249811. Information on the exact genomic position of the various BAC clones is available via **http://www.pasteur.fr/recherche/unites/Lgmb/**.

### Results

### Restriction fragment comparison of BAC

With the completion of the genome sequence of *M. tuberculosis* H37Rv [9], we have access to an *in silico* restriction map of the chromosome of the

Copyright © 2000 John Wiley & Sons, Ltd. 

highest possible resolution. Thus, restriction patterns can be predicted for a given clone, whose end-sequences are known, and compared with the experimentally derived restriction profile observed on an agarose gel. However, some of the fragments may also contain flanking vector sequences that may confound the restriction patterns. To overcome this, we developed a strategy to directly compare restriction fragments from *M. tuberculosis* H37Rv BAC clones with the corresponding fragments from BACs harbouring *M. bovis* BCG Pasteur DNA, using the same restriction endonuclease (*Hin*dIII) for restriction analysis that was used for the construction of both BAC libraries [6,12]. As such, the vector is contained in a single band and the remaining bands of the pattern correspond to the restriction fragments of the cloned mycobacterial insert. This approach allowed direct size comparison of *Hin*dIII fragments from partially or fully overlapping *M. tuberculosis* H37Rv and BCG clones and led to the identification of two duplications in the genome of *M. bovis* BCG Pasteur.

### DU1

DU1 was first observed when the banding pattern of *Hin*dIII digests from BCG BAC clone X038 and H37Rv-BAC clone Rv13 were compared to each other. Clones X038 and Rv13 have identical end-sequences, stretching from the *Hin*dIII site at genomic position 4367 kb via 4412 kb to another *Hin*dIII site at 27 kb, and thus encompass the origin of chromosomal replication (*oriC*). *In silico* analysis of the *Hin*dIII restriction sites for this stretch revealed one *Hin*dIII site at position 4404 kb in the *M. tuberculosis* H37Rv genome sequence. Therefore, the digests of these clones should contain two large restriction fragments plus the vector-specific band at 8 kb. This was the case for the *M. tuberculosis* H37Rv clone Rv13. In contrast, BCG BAC clone X038 exhibited three bands (plus the 8 kb fragment), two of which were identical to those obtained from Rv13 (Figures 1 and 2). The additional band was ~29 kb in size. Further PFGE analysis using the endonuclease *Dra*I revealed that X038 was indeed 29 kb larger than Rv13 (data not shown). By PCR screening pools of BCG BACs using selected oligonucleotides, we identified three more X-clones covering parts of this genomic region in BCG: X585, X592 and X703. End-sequence and PFGE analysis showed that each clone contained one of the three bands seen in the X038 digest (Figures 1 and 2).

The end-sequences for the BCG BACs were: X585 (4367–4404 kb); X592 (4404–4404 kb); and X703 (4404–27 kb). The puzzling result that clone X592 had both endsequences in the same genomic region could be explained by a direct duplication in BCG and this also gave us an indication of the size of the rearrangement. Further restriction and sequence analysis allowed precise identification of the junction.

PCR with primers corresponding to genomic positions 16 000 bp and 4 398 700 bp (Table 1) gave a product of the expected size when clone X592 or genomic DNA of BCG Pasteur were used as templates. Direct sequencing of the PCR products and the BAC clone X592 revealed that the junction was located between positions 16 732 and 4 398 593 relative to the *M. tuberculosis* H37Rv genomic sequence, and that this genomic rearrangement resulted in the truncation of genes *Rv3910* and *pknB*. However, as the rearrangement is a tandem duplication, intact copies of both genes should be present in the neighbouring regions. PCR analysis with flanking primers for *Rv3910* and *pknB* confirmed this when genomic DNA from BCG Pasteur and *M. tuberculosis* H37Rv were employed (data not shown).

Definitive confirmation of the rearrangement was obtained by hybridizing a 0.5 kb radio-labelled probe encompassing the *oriC* region of *M. tuberculosis* H37Rv to *Hin*dIII digests of genomic DNA of *M. tuberculosis*, *M. bovis* and BCG Pasteur. A single band of ~35 kb was detected in the *M. bovis* and *M. tuberculosis* genomes, whereas the probe hybridized to two DNA fragments, one of which was 35 kb, and the other 29 kb (Figure 1) when BCG Pasteur was examined. In summary, DU1 corresponds to a tandem duplication of 29 668 bp resulting in merodiploidy for the region *sigM–pabA* (*Rv3911–Rv0013* [9]).

### Analysis of the *dnaA–dnaN* region in strains with DU1

The *dnaA–dnaN* region is generally regarded as the functional origin of replication for mycobacteria since, on insertion into plasmids lacking their own origin of replication, the ability to replicate autonomously was restored. Evidence for this was first obtained in *M. smegmatis* [22] and, more recently,

Copyright © 2000 John Wiley & Sons, Ltd. 

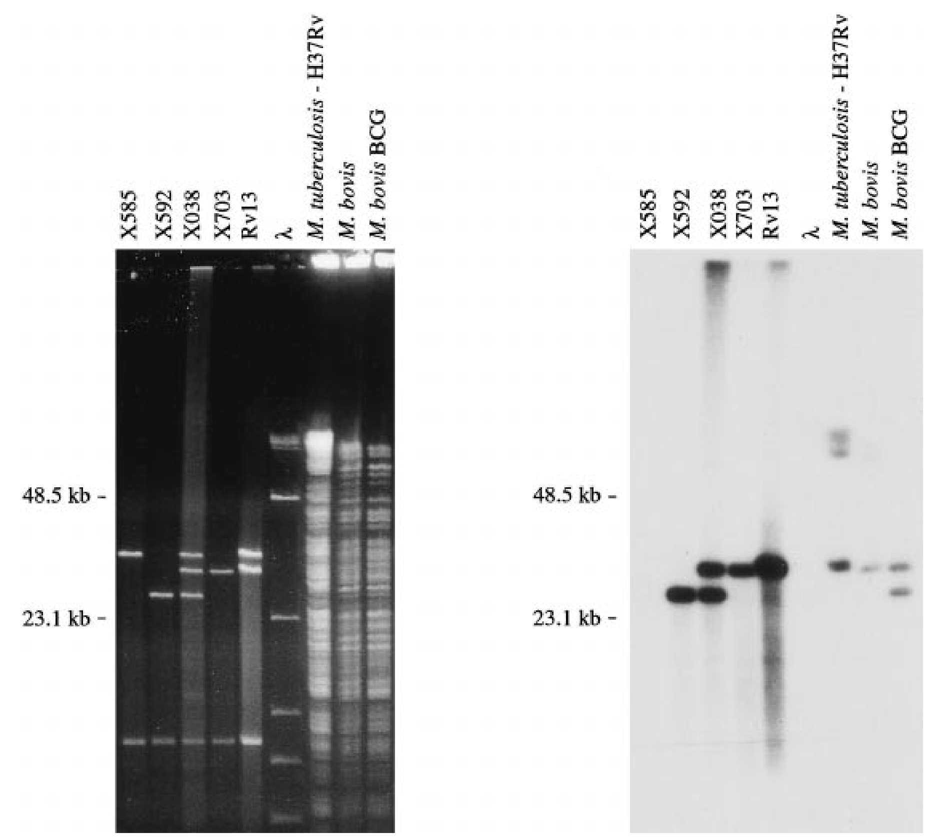

**Figure 1.** Detection of tandem duplication DU1. *Hin*dIII digestion of *M. tuberculosis* H37Rv (Rv) and BCG (X) BAC clones, *M. tuberculosis* H37Rv, *M. bovis* type strain ATCC 19210 and BCG Pasteur genomic DNA and pulsed field gel electrophoresis (1 s ramped to 2 s over 24 h, 1% agarose gel) reveal an additional genomic fragment of ~29 kb in clone X038, not present in Rv13. Radio-labelled 0.5 kb probe from the *oriC* genomic region binds to one fragment in *M. tuberculosis* and *M. bovis* and to two fragments in BCG Pasteur. Molecular weight is marked in (kb)

in *M. tuberculosis* and BCG [21]. As BCG Pasteur is diploid for the *dnaA–dnaN* region, we investigated whether any nucleotides differed between the two copies present on the two different BAC clones X592 and X703 (Figures 1 and 2). Direct sequence analysis of BAC DNA using flanking and internal primers of *dnaA–dnaN* intergenic region (Table 1) revealed no differences between the two copies of the minimal *oriC* region. In addition, the sequences were identical to the one determined by Qin *et al.* (Accession No. U75298) for their strain of BCG. This finding suggests that both copies of *oriC* should be functional.

Similarly, screening of a BCG Pasteur Tn5367 [3] transposon library revealed an insertion in one of the *dnaA* genes at position 850 bp (E. Wooff, S. Gordon and R.G. Hewinson, personal communication). It was initially surprising to find a transformant harbouring a transposon in *dnaA*, as this is normally an essential gene. However, as BCG Pasteur is diploid for *dnaA*, the second functional copy of *dnaA* could compensate for the loss of *dnaA* during transposon mutagenesis.

### DU2

The second large genomic rearrangement observed in the BCG Pasteur chromosome was found on analysis of several BAC clones, corresponding to the region between 3550 and 3750 kb, since their experimentally determined sizes did not agree with those predicted from endsequence data. Direct comparisons were complicated by the presence of an IS*6110* element in this region of the *M. tuberculosis* H37Rv chromosome that has led to a small deletion, RvD5 (see below) [7].

Copyright © 2000 John Wiley & Sons, Ltd. 

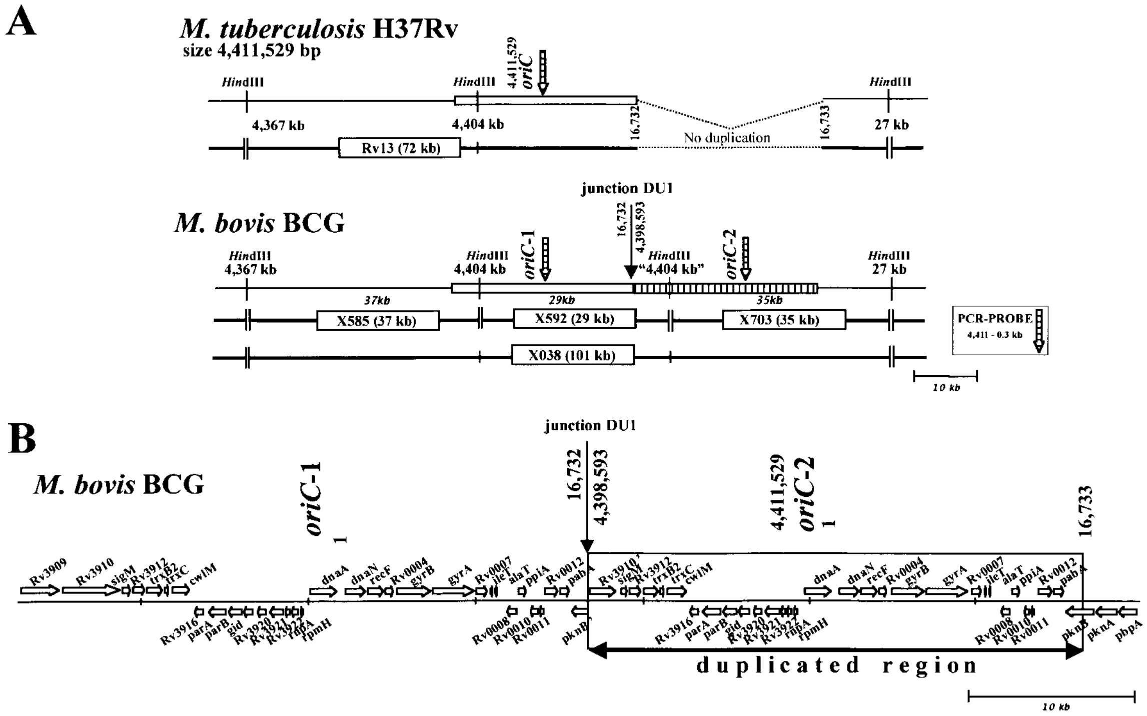

**Figure 2.** (A) Scheme of the genomic organisation in the corresponding regions encompassing the origin of replication in BCG Pasteur and *M. tuberculosis* H37Rv, revealed by BAC mapping, PCR and hybridization experiments. (B) Gene predictions in the rearranged genomic region DU1 in BCG Pasteur are based on the highly similar *M. tuberculosis* H37Rv genomic nucleotide sequence (Accession No. AL123456) and the BAC mapping data. Duplicated regions are indicated by arrows. For information about predicted gene functions, consult the website **http://bioweb.pasteur.fr/GenoList/TubercuList/**

The end-sequences of BAC clone X495 were both located around the *Hin*dIII site at 3594 kb. PFGE results showed that the clone was about 106 kb in size, containing three chromosomal *Hin*dIII fragments of 37.5, 37 and 24 kb in addition to the vector. The 24 kb band was about 2 kb larger than the corresponding 22 kb *Hin*dIII fragment in Rv403 (Figures 3 and 4). This suggested that the genomic region around 3594 kb might have been duplicated and that one junction should be near one end of clone X495. To confirm this, primer walking was performed on BAC X495 and a junction, named JDU2A, was identified between positions 3 690 124 and 3 590 900 relative to the *M. tuberculosis* H37Rv genomic sequence (Figure 4). This interrupted the gene *lpdA* (*Rv3303*), but PCR results indicated that an intact copy is present in the duplicated region (Figure 4B).

Systematic analysis of other clones in the neighbourhood identified two independent BCG BACs (X094 and X1026), which carried the same chromosomal segment, 3594–3749 kb. While the end-sequence data suggested that the clones should be about 155 kb, size estimation based on PFGE-separated *Dra*I or *Hin*dIII digestions revealed that the BACs were only ~100 kb in size. This discrepancy indicated that the inserts in clones X094 and X1026 probably extended from the repeated *Hin*dIII sites at 3594 kb to the 'authentic' *Hin*dIII site at position 3749 kb (Figure 4), and that an internal deletion had occurred within the duplicated unit.

This was confirmed by hybridization experiments on *Hin*dIII-digested genomic DNA from *M. tuberculosis* H37Rv, *M. bovis* and BCG Pasteur, using radiolabelled DNA from clone X495. Figure 3 shows that one of the bands hybridizing in the *Hin*dIII profiles of *M. tuberculosis* H37Rv and the *M. bovis* type strain was about 22 kb in size, while the corresponding band in BCG was 24 kb in size, exactly as seen with the BAC clones. Furthermore, the hybridization results showed that a band of 34 kb in the *Hin*dIII profile of clone X094 also hybridized with genomic DNA from clone X495,

Copyright © 2000 John Wiley & Sons, Ltd.

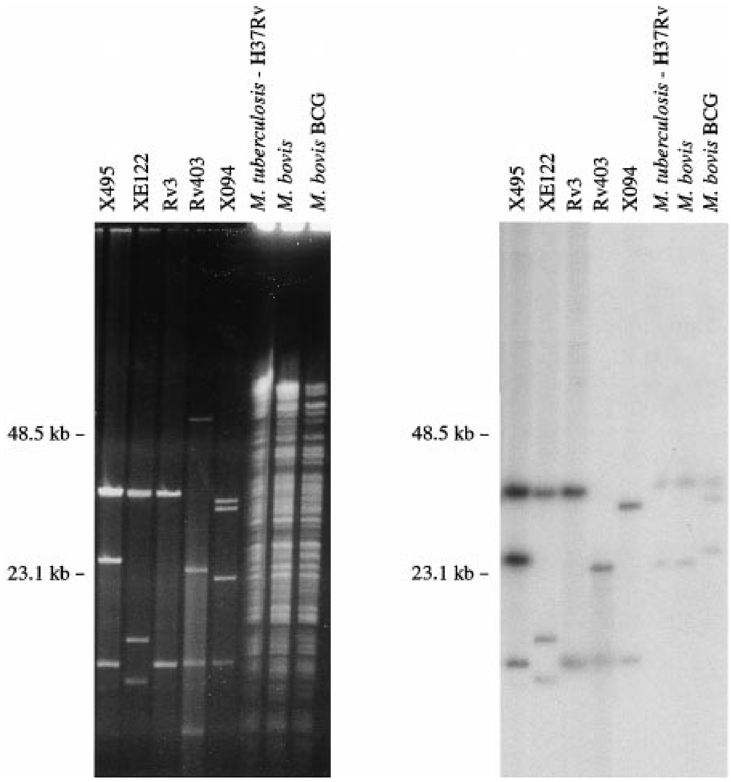

**Figure 3.** Detection of tandem duplication DU2. *Hin*dIII digestion of *M. tuberculosis* H37Rv (Rv) and BCG (X and XE) BAC clones, *M. tuberculosis* H37Rv, *M. bovis* type strain ATCC 19210 and BCG Pasteur genomic DNA and pulsed-field gel electrophoresis (1 s ramped to 2 s over 24 h, 1% agarose gel) reveal restriction fragment polymorphism in BCG- and *M. tuberculosis* BAC clones as described in Results. Radiolabelled BAC DNA from clone X495 binds to two fragments in *M. tuberculosis* and *M. bovis* and to three fragments in BCG Pasteur. Molecular weight is marked in kb confirming that clones X094 and X1026 contained duplicated DNA from the genomic region covered by X495 (Figures 3 and 4). PCR and DNA sequencing by primer walking on BAC X094 identified a second junction point, JDU2B, at a position equivalent to 3 608 471 and 3 671 535 in *M. tuberculosis* H37Rv. This confirmed that DU2 resulted from a direct duplication of the 99 225 bp region corresponding to the sequences between positions 3 590 900 and 3 690 124 in the genome of *M. tuberculosis* H37Rv, and that an internal deletion of 63 064 bp then took place. The residual DU2 unit is thus 36 162 bp in length, consistent with the mapping data, and BCG Pasteur is diploid for genes *Rv3213c–Rv3230c*, and *Rv3290c–Rv3302c* [9].

Finally, PCR, PFGE mapping and end-sequencing experiments with BAC X094 suggested that BCG Pasteur contained additional DNA in the chromosomal region of *Hin*dIII site 3691–3749. Direct comparison with the *M. tuberculosis* H37Rv BAC clone Rv403 uncovered two extra *Hin*dIII sites in this region, since the *Hin*dIII fragment of 48 kb present in Rv403 (corresponding to the segment 3691–3749; Figure 4), was represented by two bands of 22 and 36 kb in BCG. This region of the *M. tuberculosis* H37Rv chromosome contains a copy of IS*6110* that is not flanked by the characteristic 3 bp direct repeats [13,24]. It is now clear that there were originally two copies of IS*6110* that served as the substrate for a recombination event. This resulted in the deletion of a 4 kb segment from the *M. tuberculosis* H37Rv genome (RvD5) [7], which is still present in BCG, as well as in *M. bovis* and *M. tuberculosis* clinical isolates. Sequence analysis of this region has shown that this 4 kb fragment contains two *Hin*dIII sites (Figure 4) and lacks the IS*6110*, which is present at this location in *M. tuberculosis* H37Rv. Using internal primers for RvD5 (Table 1), we obtained amplicons with

Copyright © 2000 John Wiley & Sons, Ltd. 

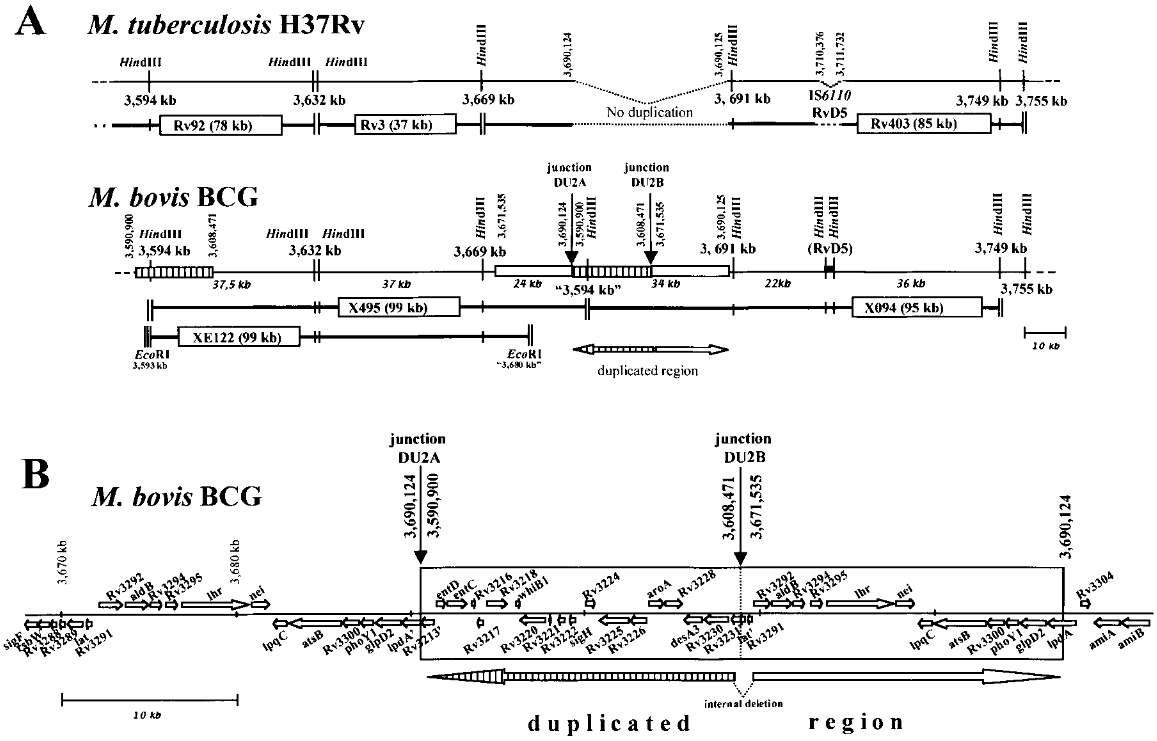

**Figure 4.** (A) Scheme of the genomic organization in the corresponding regions from 3590 kb to 3755 kb in BCG Pasteur and *M. tuberculosis* H37Rv, revealed by BAC mapping, PCR and hybridization experiments. (B) Gene predictions in the rearranged genomic region DU2 in BCG Pasteur are based on the highly similar *M. tuberculosis* H37Rv genomic nucleotide sequence (Accession No. AL123456) and the BAC mapping data. Duplicated regions are shown as boxes. For information about gene predicted functions, consult the website **http://bioweb.pasteur.fr/GenoList/TubercuList/**

genomic DNA from all of the *M. bovis* BCG substrains tested, the *M. bovis* type strain, as well as with DNA from clones X094 and X1026, but not from *M. tuberculosis* strains H37Rv and H37Ra (data not shown).

### Are some BCG cells partially triploid?

On screening 2000 X- and XE-clones [12] for BACs containing both the JDU2A and the JDU2B junctions, i.e. which covered the whole rearranged region, three BACs (X1070, XE377 and XE256) were identified that produced amplicons with both sets of primers. Their inserts were estimated to be ~95, 86 and 97 kb in length, respectively, by PFGE. On the basis of the PCR results, end-sequence data (corresponding to regions 3632 and 3594 kb), and the presence of three chromosomal *Hin*dIII fragments, of 37, 36, and 24 kb (Figure 5A, B), we concluded that clone X1070 overlaps clone X495. However, it contained a 36 kb chromosomal *Hin*dIII fragment which was present in neither clone X495 nor in clone X094 and, together with the end-sequence data, this suggested the presence of a third copy of the *Hin*dIII site at 3594 kb in the rearranged region. Further evidence for this was obtained when clones XE256 and XE377, isolated from an *Eco*RI library in pBACe3.6, were analysed. According to end-sequence data, XE256 extends from the *Eco*RI site at 3597 kb to the *Eco*RI site at 3713 kb, and XE377 from *Eco*RI site at 3679 kb to the *Eco*RI site at 3715 kb. The fact that these clones repeatedly yielded amplicons for both junction regions JDU2A and JDU2B did not agree with their size and their end-sequences. However, as shown in Figure 6, these findings were consistent with the 36 162 bp region DU2 being present as not only one but two tandem copies. Hybridization of *Hin*dIII-digested DNA from clones XE256, X1070 and XE377 with a 0.5 kb probe from genomic region 3675 kb confirmed the PCR results. As depicted in Figure 5B, one 24 kb fragment of clone

Copyright © 2000 John Wiley & Sons, Ltd. 

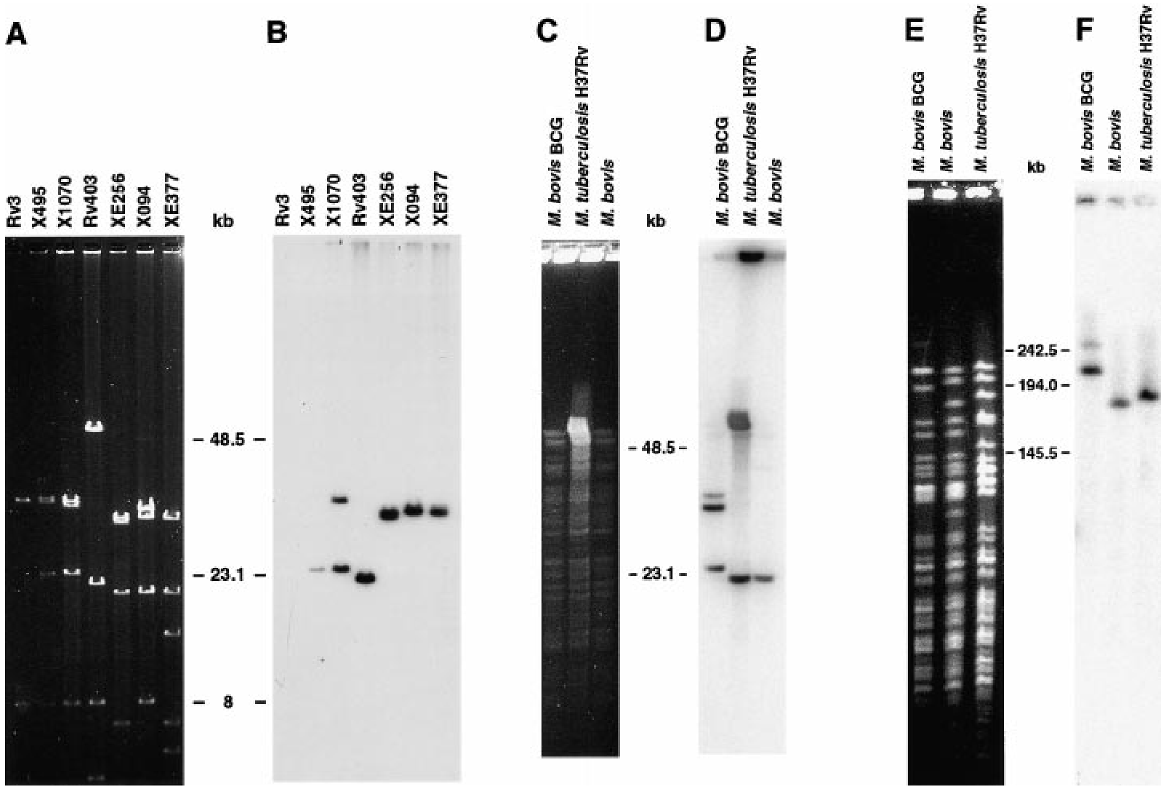

**Figure 5.** Detection of a second copy of DU2. (A) *Hin*dIII digestion of *M. tuberculosis* H37Rv (Rv) and BCG (X and XE) BAC clones and pulsed-field gel electrophoresis (1 s ramped to 1.5 s over 24 h, 1% agarose gel) reveals restriction fragment polymorphism in BCG and *M. tuberculosis* BAC clones, as described in Results. (B) Radiolabelled 0.5 kb probe from the genomic region 3675 binds to BCG and *M. tuberculosis* BAC clone fragments, as described in Results. The hybridization signal for the two hybridizing fragments in BAC clone X1070 is equally strong. (C) *Hin*dIII digestion of BCG Pasteur, *M. bovis* and *M. tuberculosis* H37Rv genomic DNA and pulsed-field gel electrophoresis (1 s ramped to 1.5 s over 24 h, 1% agarose gel) reveals restriction fragment polymorphism in BCG Pasteur, *M. bovis* and *M. tuberculosis* H37Rv, as described in Results. (D) Radiolabelled 0.5 kb probe from the genomic region 3675 binds to one fragment in *M. tuberculosis* and *M. bovis* and to three fragments in BCG Pasteur. The hybridization signals of the corresponding fragments present in the genomic DNA from BCG Pasteur show a difference in the strength of the signal. Molecular weight is indicated in kb. (E) *Xba*I digestion of BCG Pasteur, *M. bovis* type strain and *M. tuberculosis* H37Rv genomic DNA and pulsed-field gel electrophoresis (1 s ramped to 18 s over 29 h, 1% agarose gel) reveal different *Xba*I patterns. BCG exhibits a weak band of ~250 kb which is not present in the profiles of *M. tuberculosis* and *M. bovis*. (F) A radiolabelled 0.5 kb probe from the genomic region 3675 binds to one fragment in the size range of 183 kb in *M. tuberculosis* H37Rv and to the corresponding band in *M. bovis*, which is about 5 kb smaller, as the number of present IS elements is different. Probe 3675 binds to two fragments in BCG Pasteur, which are ~36 or ~72 kb larger than the corresponding 178 kb band in *M. bovis*, due to the presence of one or two copies of DU2. The hybridization signal for the 250 kb band is weaker than that of the 215 kb fragment

X1070 hybridized, equivalent to that in clone X495, and a unique 36 kb fragment, covering an additional copy of DU2, was also present. Two fragments of 33 kb and 34 kb from clone XE256 hybridized. The 33 kb fragment corresponds to the region extending from the *Hin*dIII site present in the vector, adjacent to the *Eco*RI cloning site, to the closest *Hin*dIII site in the mycobacterial insert, while the 34 kb fragment was identical to the one also present in clone X094 (Figures 5B, 6A). The 33 kb fragment overlapped in part clone X1070 while the 34 kb *Hin*dIII fragment was identical to those present in clones X094 and XE377.

These findings indicated that two tandem copies of DU2 existed in the genome of BCG Pasteur. This was confirmed on hybridization of *Hin*dIII digests

Copyright © 2000 John Wiley & Sons, Ltd.

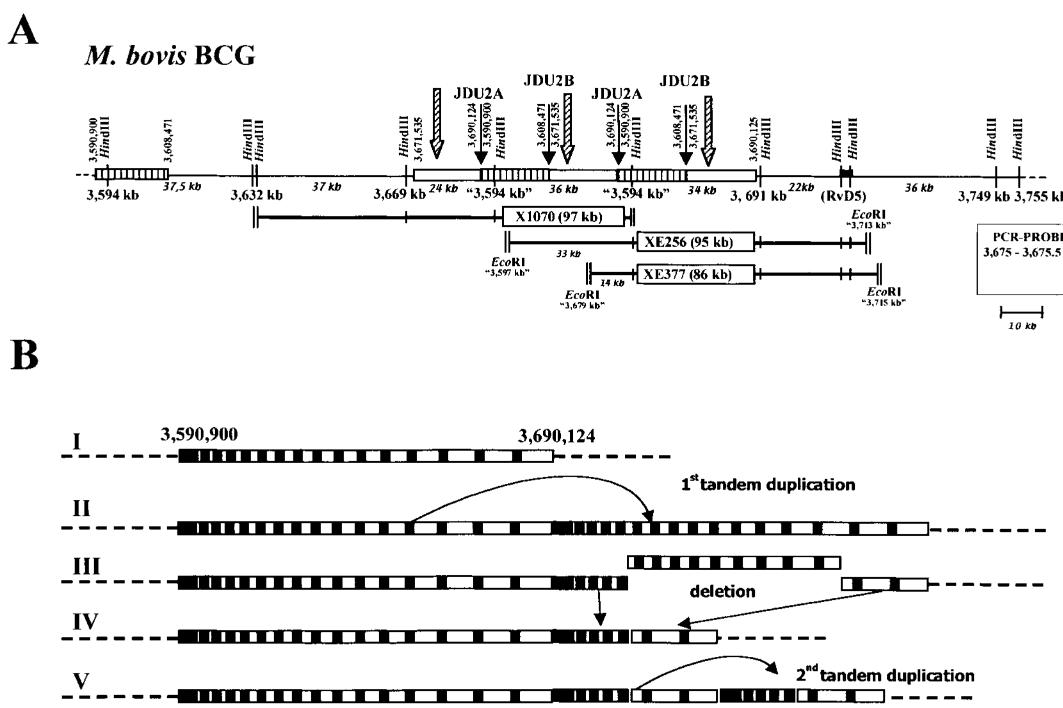

**Figure 6.** (A) Scheme of the genomic organization in the corresponding regions from 3590 kb to 3755 kb in a subpopulation of BCG Pasteur containing a second copy of DU2, revealed by BAC mapping, PCR and hybridization experiments. Duplicated regions are shown as boxes. (B) Scheme of the putative mechanism which led to the appearence of DU2 in BCG Pasteur of genomic DNA from BCG Pasteur, *M. tuberculosis* H37Rv and *M. bovis*, since these all hybridized with probe 3675. As expected, only one 22 kb band was observed with *M. tuberculosis* and *M. bovis*, while three bands of 24, 34 and 36 kb were detected in the hybridization pattern of BCG Pasteur. However, as shown in Figure 5D, the hybridization signal for the 36 kb fragment was very weak. As the 24 and 36 kb bands present in BAC clone X1070 hybridized to probe 3675 with the same intensity (Figure 5B), whereas those in the genomic DNA of BCG Pasteur did not (Figure 5D), this suggested that only a subpopulation of the BCG Pasteur culture contained the second copy of DU2. Hence, the difference seen in the intensity of hybridization may reflect the second copy of DU2 having been gained only recently, and indicates that variants containing one or two copies of DU2 exist in the same tube of lyophylized *M. bovis* BCG Pasteur 1173 vaccine. In order to make sure that the BCG Pasteur culture in use was not contaminated with other BCG substrains, which may have accounted for the extra band observed, we performed PCR experiments using oligonucleotides (Table 1) that are specific for a genomic region that is absent in BCG Pasteur 1173, but present in all other BCG substrains [2]. PCR did not yield any amplicons with the BCG Pasteur DNA, while controls with other BCG substrains (Danish, Glaxo) and oligonucleotides from other genomic regions yielded amplicons of the appropriate sizes (data not shown).

Further evidence for the presence of the two variants were obtained when *Xba*I digests of genomic DNA from *M. tuberculosis*, *M. bovis*, and BCG Pasteur were hybridized with probe 3675 (Figure 5E, 5F). In the *M. tuberculosis* H37Rv digest, probe 3675 hybridized to a 183 kb fragment (genomic position 3646–3829 kb). The corresponding fragment in the *M. bovis* type strain was ~178 kb and this size difference is due to the absence of several insertion elements which are only present in the 183 kb genomic region in *M. tuberculosis* H37Rv. The *Xba*I digest of BCG Pasteur contained two fragments, of 215 and 250 kb, which hybridized to probe 3675. These correspond to the 178 kb fragment seen in the *M. bovis* genome, increased by either 36 or 72 kb due to

Copyright © 2000 John Wiley & Sons, Ltd. 

the presence of one or two copies of DU2. It is noteworthy that the hybridization signal for the 250 kb fragment was less intense than the signal obtained with the 215 kb fragment, confirming previous observations with *Hin*dIII digests. These findings indicate that this region of the BCG genome is still dynamic and that a subpopulation of cells are triploid for *Rv3213c–Rv3230c*, and *Rv3290c–Rv3302c* [9].

### Screening for duplications in various BCG substrains

Preliminary PCR and PFGE analyses in various BCG substrains, using primers JDU1, JDU2A and JDU2B (Table 1), suggested that DU1 is present only in BCG Pasteur (data not shown), while DU2 is present in all tested BCG strains but at variable sizes (data not shown). However, before drawing more conclusions, a complete set of the various daughter strains has to be screened for DU1 and DU2. Analyses of DU1 and DU2 in the other BCG daughter strains using primers that flank the junction regions, as well as PFGE comparisons, are ongoing and will be the subject of a future publication.

### Discussion

The current BCG vaccines are all derived from a strain of *M. bovis* that was attenuated by 230 serial passages on potato-glycerin-ox bile medium [4,8]. Yet, despite decades of use worldwide, the reason for the attenuation of *M. bovis* BCG is still unknown, although comparative genomics is beginning to shed light on some of the possible mechanisms involved. It has been shown recently, in whole-genome scans using either BAC arrays [12] or DNA microarrays [2], that up to 16 genomic deletions can be found in BCG substrains relative to *M. tuberculosis* H37Rv. We are presently investigating what biological role these deletions play in terms of virulence and host specificity. The fact that most of the end-points of the 16 variable regions are located inside genes that show homology to genes of other organisms suggests that these regions represent deletions in BCG strains, rather than insertions in *M. tuberculosis*.

However, as array techniques are only able to detect DNA fragments which do not hybridize with the matrix DNA, duplications or other chromosomal rearrangements cannot be uncovered. Direct comparison of *M. tuberculosis* H37Rv- and BCG BACs thus represents a powerful approach for the detection of additional genomic differences, and is providing further insight into genome plasticity as well as identifying genes and gene products that may be involved in mycobacterial virulence. In the present study, the existence of two duplicated segments of the genome of BCG Pasteur, DU1 and DU2, has been demonstrated.

Inspection of the genome sequence of *M. tuberculosis* H37Rv [9] indicates that BCG Pasteur should be at least diploid for 58 genes, and that at one point their common ancestor contained duplicate copies of a further 60 genes that were lost when the deletion internal to DU2 arose. Tandem duplications are generally caused by unequal crossing over between homologous sequences, such as IS elements or *rrn* operons [15], or by recombination of short DNA homologies [10].

The mechanism responsible for the duplication of DU1 and DU2 in *M. bovis* BCG Pasteur is obscure, although one can exclude a role for IS elements, as none is found in these regions of the BCG genome [9,12]. Furthermore, no highly conserved sequences could be detected in the corresponding regions of the *M. tuberculosis* H37Rv genome [9], as is probably also the case for *M. bovis* BCG. The finding that DU2 resulted from direct duplication of a 99 225 bp segment, from which 63 064 bp were then deleted to leave a residual 36 162 bp fragment, which was then again duplicated in a subpopulation of BCG Pasteur, shows how complex duplication events may be. Dynamic duplication of large chromosomal sections were also observed in some strains of *Bordetella pertussis* (C. Weber, personal communication), suggesting that tandem duplications represent an important mechanism for generating genomic plasticity in other bacteria as well.

Chromosomal duplications provide a means for increasing gene dosage, for generating novel functions from potential gene fusion events at duplication endpoints and represent a source of redundant DNA for divergence. Over half of the proteins present in the tubercle bacillus have arisen from ancient gene duplication and adaptation events [9,23]. The presence of DU1 and DU2, and particularly the finding that DU2 appears to be present in a subpopulation of BCG Pasteur in two copies, suggests that the process of tandem duplica-

Copyright © 2000 John Wiley & Sons, Ltd.

tions in BCG is ongoing and remains a potent source of genome dynamics.

From a practical point of view, the presence of large repeated regions in the chromosome of *M. bovis* BCG underlines the importance of BAC mapping strategies to accompany genome sequencing, and highlights the potential pitfalls of projects based solely on sequencing shotgun clones. Without the BAC resource, it is very likely that the rather complex genomic rearrangements present in *M. bovis* BCG strains would have remained undetected. Knowledge of large tandem duplications is useful in interpreting previously published data. As shown in Figure 5E, a 250 kb band in the *Xba*I digest of BCG strains indicates the presence of two copies of DU2 in the genome of BCG. As this band is unique to BCG, it could be used as a marker for detecting BCG strains which carry two copies of DU2. When comparing the *Xba*I patterns of various BCG strains shown in previous work by Zhang *et al.* [26], it can be noted that the Glaxo and the Danish BCG strains carried this fragment, while others, like BCG Tice or BCG Connaught, did not.

DU1 represents a particularly interesting example of a tandem genetic duplication because it results in a strain with two copies of *oriC*, the site of initiation of chromosomal replication, and genes encoding key components of the replication initiation and cell division machinery [22]. To our knowledge, this is the first description of a bacterium with two *oriC*s. Multiple copies of *oriC* are generally deleterious for the cell, as they can provoke uncoordinated replication [22]. Although sequence comparison of the two copies of the *dnaA–dnaN* intergenic region, known to be the functional *oriC* of *M. tuberculosis* and BCG [21], revealed no differences, it is still not known whether both copies of the BCG Pasteur origin are functional *in vivo*.

It is probable that increased expression may result from some of the duplicated or triplicated genes and lead to phenotypic and immunological differences. As such effects have been described for several BCG strains [1,14], and even suggested to account for variability in vaccine efficacy [4,11], it was of interest to examine the nature of the sequences present in DU1 and DU2. Among the known genes in DU1, in addition to those involved in chromosome replication and cell division, are coding sequences for DNA gyrase, two tRNAs, the $\sigma$-factor, SigM, and a thioredoxin-thioredoxin reductase system [9]. The last three genes are particularly noteworthy, since it has been shown in *Streptomyces coelicolor* that the SigM counterpart, SigR, initiates transcription of the adjacent thioredoxin system in response to oxidative stress [18]. Furthermore, expression of the Trx system of *Mycobacterium leprae* in *Mycobacterium smegmatis* renders this avirulent saprophyte resistant to the oxygen-dependent killing mediated by human mononuclear phagocytes [25]. Genes located in DU2 for which probable functions are known include *aroA*, encoding 3-phosphoshikimate 1-carboxyvinyl transferase, a key enzyme in aromatic amino acid biosynthesis, and the coding sequences for a variety of regulatory proteins that could exert pleiotropic effects, including a histidine kinase, AsnC and TetR homologues, PhoY1, WhiB1, and another factor, sigma H.

Although at present it is difficult to speculate about the impact of multiple copies of these genes on the biology, the attenuation process and/or the immunogenicity of BCG, knowledge of their existence will facilitate quality control of BCG vaccine lots and may help in monitoring the efficacy of these vaccines.

### Acknowledgements

We thank Marcel Behr for advice and discussion. This work received financial support from the BIOMED programme of the European Community (BMH4-CT97-2277), the Wellcome Trust, the Association Française Raoul Follereau and the Institut Pasteur.

### References

1. Behr MA, Small PM. A historical and molecular phylogeny of BCG strains. *Vaccine* 1999; **17**: 915–922.
2. Behr MA, Wilson MA, Gill WP, Salamon H, Schoolnik GK, Rane S, Small PM. Comparative genomics of BCG vaccines by whole-genome DNA microarrays. *Science* 1999; **284**: 1520–1523.
3. Bardarov S, Kriakov J, Carriere C, Yu S, Vaamonde C, McAdam RA, Bloom BR, Hatfull GF, Jacobs WR Jr. Conditionally replicating mycobacteriophages: a system for transposon delivery to *Mycobacterium tuberculosis*. *Proc Natl Acad Sci U S A* 1997; **94**: 10961–10966.
4. Bloom BR, Fine PEM. The BCG experience: implications for future vaccines against tuberculosis. In *Tuberculosis: Pathogenesis, Protection and Control*, Bloom BR (ed.). American Society for Microbiology: Washington DC; 531–557.
5. Brosch R, Chen J, Luchansky JB. Pulsed-field fingerprinting of listeriae: identification of two genomic divisions for *L.*

Copyright © 2000 John Wiley & Sons, Ltd. 

*monocytogenes* and their correlation with serovar. *Appl Environ Microbiol* 1994; **60**: 2584–2592.

6. Brosch R, Gordon SV, Billault A, Garnier T, Eiglmeier K, Soravito C, Barrell BG, Cole ST. Use of a *Mycobacterium tuberculosis* H37Rv bacterial artificial chromosome (BAC) library for genome mapping, sequencing and comparative genomics. *Infect Immun* 1998; **66**: 2221–2229.
7. Brosch R, Philipp W, Stavropolous E, Colston MJ, Cole ST, Gordon SV. Genomic analysis reveals variation between *Mycobacterium tuberculosis* H37Rv and the attenuated *M. tuberculosis* H37Ra. *Infect Immun* 1999; **67**: 5768–5774.
8. Calmette A. *La Vaccination Contre la Tuberculose*. Masson: Paris; 250.
9. Cole ST, Brosch R, Parkhill J, Garnier T, Churcher C, Harris D, Gordon SV, Eiglmeier K, Gas S, Barry CE III, Tekaia F, Badcock K, Basham D, Brown D, Chillingworth T, Connor R, Davies R, Devlin K, Feltwell T, Gentles S, Hamlin N, Holroyd S, Hornsby T, Jagels K, Krogh A, McLean A, Moule S, Murphy L, Oliver K, Osborne J, Quail MA, Rajandream MA, Rogers J, Rutter S, Seeger K, Skelton J, Squares R, Squares S, Sulston JE, Taylor K, Whitehead S, Barrell BG. Deciphering the biology of *Mycobacterium tuberculosis* from the complete genome sequence. *Nature* 1998; **393**: 537–544.
10. Edlund T, Normark S. Recombination between short DNA homologies causes tandem duplication. *Nature* 1981; **292**: 269–271.
11. Fine PEM. Variation in protection by BCG: implications of and for heterologous immunity. *Lancet* 1995; **346**: 1339–1345.
12. Gordon SV, Brosch R, Billault A, Garnier T, Eiglmeier K, Cole ST. Identification of variable regions in the genomes of tubercle bacilli using bacterial artificial chromosome arrays. *Mol Microbiol* 1999; **32**: 643–656.
13. Gordon SV, Heym B, Parkhill J, Barrell B, Cole ST. New insertion sequences and a novel repeated sequence in the genome of *Mycobacterium tuberculosis* H37Rv. *Microbiology* 1999; **145**: 881–892.
14. Lagranderie MR, Balazuc AM, Deriaud E, Leclerc CD, Gheorghiu M. Comparison of immune responses of mice immunized with five different *Mycobacterium bovis* BCG vaccine strains. *Infect Immun* 1996; **64**: 1–9.
15. Lupski JR, Roth JR, Weinstock GM. Chromosomal duplications in bacteria, fruit flies, and humans. *Am J Hum Genet* 1996; **58**: 21–27.
16. Mahairas GG, Sabo PJ, Hickey MJ, Singh DC, Stover CK. Molecular analysis of genetic differences between *Mycobacterium bovis* BCG and virulent *M. bovis*. *J Bacteriol* 1996; **178**: 1274–1282.
17. Oettinger T, Jorgensen M, Ladefoged A, Haslov K, Andersen P. Development of the *Mycobacterium bovis* BCG vaccine: review of the historical and biochemical evidence for a genealogical tree. *Tubercul Lung Dis* 1999; **79**: 243–250.
18. Paget MS, Kang JG, Roe JH, Buttner MJ. SigmaR, an RNA polymerase sigma factor that modulates expression of the thioredoxin system in response to oxidative stress in *Streptomyces coelicolor* A3(2). *EMBO J* 1998; **17**: 5776–5782.
19. Philipp WJ, Nair S, Guglielmi G, Lagranderie M, Gicquel B, Cole ST. Physical mapping of *Mycobacterium bovis* BCG Pasteur reveals differences from the genome map of *Mycobacterium tuberculosis H37Rv* and from *Mycobacterium bovis*. *Microbiology* 1996; **142**: 3135–3145.
20. Philipp WJ, Poulet S, Eiglmeier K, Pascopella L, Subramanian B, Heym B, Bergh S, Bloom BR, Jacobs WR Jr, Cole ST. An integrated map of the genome of the tubercle bacillus, *Mycobacterium tuberculosis* H37Rv, and comparison with *Mycobacterium leprae*. *Proc Natl Acad Sci U S A* 1996; **93**: 3132–3137.
21. Qin MH, Madiraju MV, Rajagopalan M. Characterization of the functional replication origin of *Mycobacterium tuberculosis*. *Gene* 1999; **233**: 121–130.
22. Salazar L, Fsihi H, de Rossi E, Riccardi G, Rios C, Cole ST, Takiff HE. Organization of the origins of replication of the chromosomes of *M. smegmatis*, *M. leprae* and *Mycobacterium tuberculosis* and isolation of a functional origin from *M. smegmatis*. *Mol Microbiol* 1996; **20**: 283–293.
23. Tekaia F, Gordon SV, Garnier T, Brosch R, Barrell BG, Cole ST. Analysis of the proteome of *Mycobacterium tuberculosis in vitro*. *Tubercul Lung Dis* 1999; **79**: 329–342.
24. Thierry D, Cave MD, Eisenach KD, Crawford JT, Bates JH, Gicquel B, Guesdon JL. IS*6110*, an IS-like element of *Mycobacterium tuberculosis*. *Nucleic Acids Res* 1990; **18**: 188
25. Wieles B, Ottenhoff TH, Steenwijk TM, Franken KL, de Vries RR, Langermans JA. Increased intracellular survival of *Mycobacterium smegmatis* containing the *Mycobacterium leprae* thioredoxin-thioredoxin reductase gene. *Infect Immun* 1997; **65**: 2537–2541.
26. Zhang Y, Wallace RJ Jr, Mazurek GH. Genetic differences between BCG substrains. *Tubercul Lung Dis* 1995; **76**: 43–50.

Copyright © 2000 John Wiley & Sons, Ltd.
